# Mesenchymal Stromal Cell-Derived Extracellular Vesicles Regulate the Mitochondrial Metabolism *via* Transfer of miRNAs

**DOI:** 10.3389/fimmu.2021.623973

**Published:** 2021-03-16

**Authors:** Claire Loussouarn, Yves-Marie Pers, Claire Bony, Christian Jorgensen, Danièle Noël

**Affiliations:** ^1^ IRMB, University of Montpellier, INSERM, CHU Montpellier, Montpellier, France; ^2^ Clinical Immunology and Osteoarticular Diseases Therapeutic Unit, Department of Rheumatology, Lapeyronie University Hospital, Montpellier, France

**Keywords:** mesenchymal stem cell, miRNA, mitochondrial metabolism, extracellular vesicle, mitochondrial dysfunction

## Abstract

Mesenchymal stromal cells (MSCs) are the most commonly tested adult progenitor cells in regenerative medicine. They stimulate tissue repair primarily through the secretion of immune-regulatory and pro-regenerative factors. There is increasing evidence that most of these factors are carried on extracellular vesicles (EVs) that are released by MSCs, either spontaneously or after activation. Exosomes and microvesicles are the most investigated types of EVs that act through uptake by target cells and cargo release inside the cytoplasm or through interactions with receptors expressed on target cells to stimulate downstream intracellular pathways. They convey different types of molecules, including proteins, lipids and acid nucleics among which, miRNAs are the most widely studied. The cargo of EVs can be impacted by the culture or environmental conditions that MSCs encounter and by changes in the energy metabolism that regulate the functional properties of MSCs. On the other hand, MSC-derived EVs are also reported to impact the metabolism of target cells. In the present review, we discuss the role of MSC-EVs in the regulation of the energy metabolism and oxidative stress of target cells and tissues with a focus on the role of miRNAs.

## Introduction

Mitochondria are complex organelles that play a central role in energy metabolism, biosynthetic processes and control of stress responses. Mitochondrial function or ability to generate energy through OXPHOS (oxidative phosphorylation) is vital for cell homeostasis. Mitochondrial dysfunctions are a hallmark of many diseases including metabolic disorders, cardiomyopathies, neurodegenerative diseases and cancer, tightly associated with programmed cell death (apoptosis). The regulation of mitochondrial metabolism is therefore essential to maintain tissue homeostasis (1).

Mesenchymal stromal cells (MSCs) have been shown to impact mitochondrial function [for review, see (2)]. MSCs are multipotent adult progenitor cells first identified in the bone marrow (BM) (3). In addition to BM, MSCs have been described and isolated from several tissues, including adipose tissue, umbilical cord, placenta, or dental pulp (4). The International Society for Cellular Therapy (ISCT) proposes three criteria to define MSCs: i) their adherence to plastic, ii) their immunophenotype CD105^+^, CD73^+^, CD90^+^, and CD45^-^, CD34^-^, CD14^-^, or CD11b^-^, CD79a^-^ or CD19^-^, HLA-DR^-^, and iii) their capacity to differentiate into osteoblasts, adipocytes and chondrocytes (5). MSCs express varying levels of tissue factor (TF/CD142) depending on the tissue source, which may trigger instant blood-mediated inflammatory reaction (IBMIR) and should be checked before clinical application when intravascular delivery is intended (6). MSCs are also characterized by their paracrine function: i) they support survival and differentiation of hematopoietic stromal cells, ii) induce cell proliferation and iii) have anti-fibrotic, anti-apoptotic, pro-angiogenic, anti-bacterial and anti-inflammatory functions (7). Although MSCs from different tissue sources share similar properties, they may display differences in their differentiation potential or trophic capacity (8, 9). They are the most commonly used cells in tissue engineering and regenerative medicine by promoting tissue repair through different mechanisms and function by both cell contact-dependent and independent mechanisms. It is generally assumed that a large share of their effector function is primarily mediated through both cell surface presentation or extracellular secretion of both cell-bound and soluble trophic and immunomodulatory factors (10, 11). These factors can be secreted as single factors or contained within extracellular vesicles (EVs), which are loaded with a complex cargo and mediate their effector functions after entrapment by target cells (12).

EVs are a heterogeneous family of vesicles characterized by a lipid bilayer. On the basis of their biogenesis and size, they are classified into three major subtypes: exosomes (<150 nm) released from the endosomal compartment, microvesicles or microparticles (>150 nm) produced by budding from the plasma membrane and apoptotic bodies originating from disassembling apoptotic cells (13). EVs are major actors in extracellular communication *via* the delivery of their cargo either by fusion with the plasma membrane of target cells or by endocytosis and release into the cytosol (14). EVs contains proteins, lipids and nucleic acids, including mRNA, lncRNA, miRNA, whose identity and quantity vary according to the parental cell and the physiologic environment [for review, see (15)]. MiRNAs are small noncoding RNAs acting as post-transcriptional regulators of gene expression. The role of miRNAs in the therapeutic function of MSC-derived EVs has been widely investigated and demonstrated in a number of diseases [for review, see (16)]. miRNA can target genes involved in mitochondrial function. In this review, we propose to discuss the impact of MSC-EVs on mitochondrial metabolism and the role played by miRNAs in this regulation.

## Mitochondrial Metabolism

### The Mitochondrial Organelle: Structure and Function

**Figure 1:**
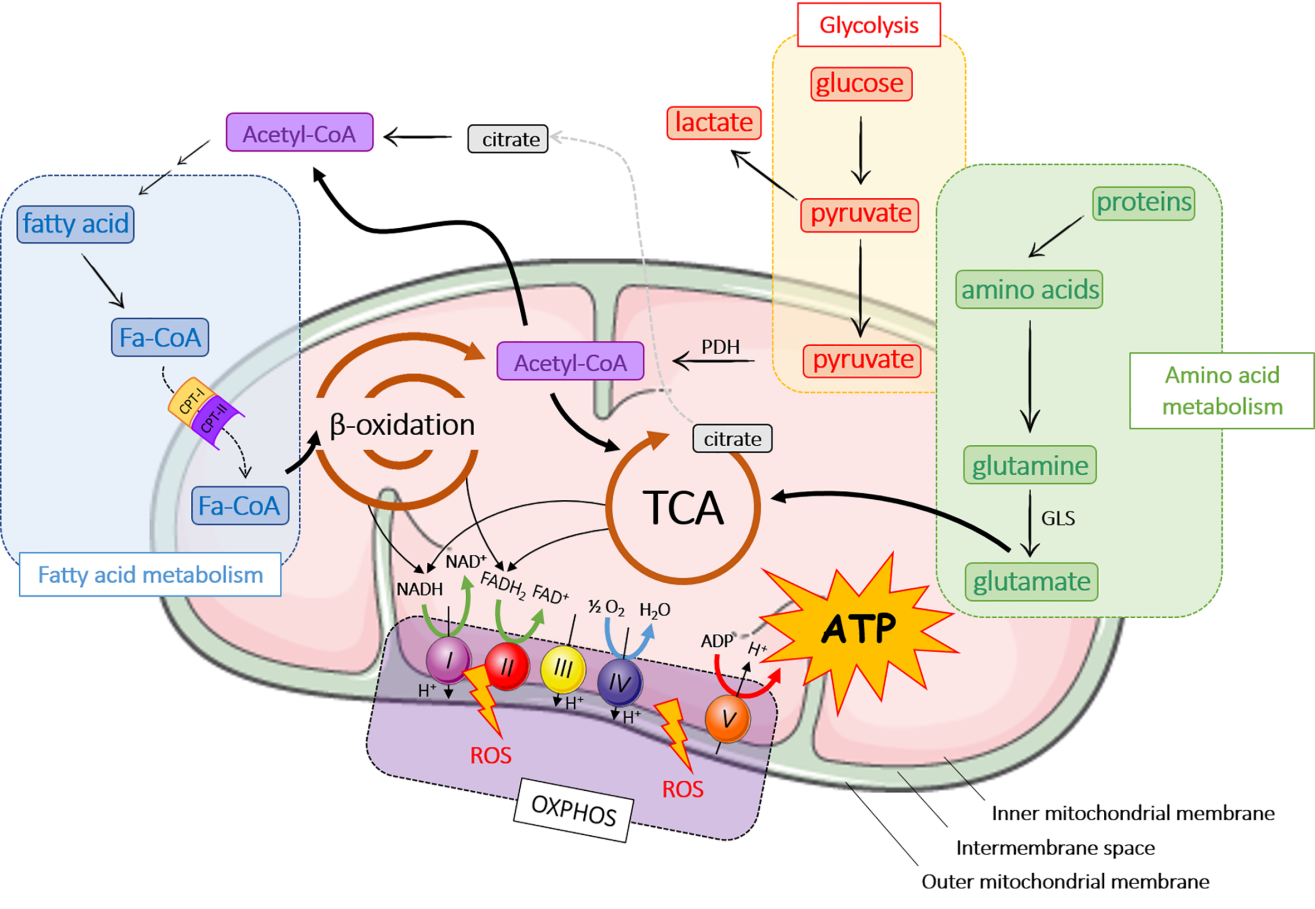
Major metabolic pathways in mitochondria. ADP, adenosine diphosphate; ATP, adenosine triphosphate; CPT, carnitine palmitoyltransferase; FADH2, flavin adenine dinucleotide; GLS, glutaminase; OXPHOS, oxidative phosphorylation; NADH, nicotinamide adenine dinucleotide hydrogenase; PDH, pyruvate dehydrogenase; ROS, reactive oxygen species; TCA, tricarboxylic acid cycle.

Mitochondria are found in most eukaryotic cells and are the cell energy-producing organelles. There are up to 2,000 per cell (according to cell type), and are preferentially located in adenosine triphosphate (ATP)-consuming cellular areas. They are small organelles (0.5 to 1 µM) surrounded by a double membrane; each membrane being composed of a phospholipid bilayer (17). The two membranes, the outer (OMM) and the inner mitochondrial membrane (INM), delimit three media: the extra-mitochondrial medium (cytoplasm of the cell), the mitochondrial intermembrane space (IMS), and the mitochondrial matrix (**Figure 1**).

Mitochondria contribute to the cellular respiration processes through ATP production. ATP is the ubiquitous energy molecule used in a very large number of cell metabolic reactions [for review, see (18, 19)]. Apart from their involvement in ATP production, mitochondria play also a key role in cell signalling, differentiation, cell death, and control of cell growth (20). Mitochondria have their own genome, transcription and translation systems, but they also require proteins encoded by the nucleus to be functionally active (21). Mitochondria are therefore in the core of several biological processes, but are also involved in mitochondrial dysregulation-based diseases.

### Mitochondrial Metabolic Pathways

The main role of mitochondria is to produce energy through ATP release following a series of chemical reactions, better known as the TriCarboxylic Acid cycle (TCA) or Krebs cycle (**Figure 1**). ATP is also generated from fatty acid metabolism and β-oxidation, OXPHOS and amino acid metabolism (**Figure 1**). In aerobic organisms, cellular energy mostly derived from glycolysis that converts glucose into pyruvate in the cytoplasm. Pyruvate enters mitochondria where it is converted into acetyl-CoA by pyruvate dehydrogenase (PDH) and then into citrate during the first step of TCA. TCA cycle is composed of ten steps catalyzed by eight different enzymes that produce nicotinamide adenine dinucleotide hydrogenase (NADH) and flavin adenine dinucleotide (FADH2) that are oxidized by the respiratory chain or OXPHOS to produce ATP. OXPHOS consists of the electron transport chain (ETC), which includes a series of protein complexes in the IMS: NADH-dehydrogenase (complex I), succinate dehydrogenase (complex II), ubiquinone, *bc*1 complex (complex III), cytochrome c and cytochrome c oxidase (CYTC, CCO; complex IV) and ATP synthase (complex V). ETC generates a proton gradient, which drives the synthesis of ATP. During OXPHOS process, mitochondria generate reactive oxygen species (ROS), which participate to cell homeostasis, primarily by the genesis of hypoxia-inducible factors (HIF) (22–24). However, ROS accumulation induces mitochondrial damage and dysfunction leading to cellular modifications.

A part from glycolysis, acetyl-CoA can be produced by the breakdown of lipids (β-oxidation) or proteins (amino acid metabolism) (25). Fatty acid oxidation occurs in mitochondria after fatty acids have crossed mitochondrial membranes under the form of fatty acyl-CoA (Fa-CoA) that is converted into acyl-carnitine by carnitine palmitoyltransferase 1 (CPT-1) in the OMM (26). In the mitochondria, carnitine palmitoyl transferase 2 (CPT-2) removes carnitine from the acyl-carnitine and regenerates acetyl-CoA. During β-oxidation, Fa-CoAs are subsequently cleaved into two carbon segments to synthesize acetyl-CoA. As the final product of β-oxidation, acetyl-CoA takes part in other reactions, primarily TCA cycle and *de novo* lipid synthesis.

Several pathways of the amino acid metabolism are located in mitochondria. Amino acids are either ketogenic (lysine and leucine), glucogenic (glycine, serine, glutamine…), or both (tyrosine, tryptophan…). Depending on their carbon skeleton, ketogenic amino acids are converted into acetyl-CoA or acetoacetyl-CoA, while glucogenic amino acids are converted into glucose, pyruvate or a TCA cycle intermediate. In this pathway, glutaminase converts glutamine to glutamate, which is further catabolized in TCA cycle (21).

### Mitochondria-Related Pathologies

The role of mitochondria in multiple other functions, notably Ca^2+^ homeostasis, ROS generation and apoptosis, has been shown (27). Mitochondria are therefore in the centre of cell homeostasis and their dysfunctions are associated to pathological conditions (28, 29). Mitochondrial disorders are characterized by defective oxidative phosphorylation and mainly observed in energy-dependent tissues such as skeletal muscle, heart, peripheral, and central nervous system, eyes, kidney or endocrine glands. Dysfunctions include depolarization, ETC inhibition and network fragmentation that impact secretion of metabolites, ROS production and affect cell signalling pathways. Main causes are related to mitochondrial or genomic DNA mutations, which alter the synthesis of ETC enzymatic complexes (30), endoplasmic reticulum stress (31), supercomplex destabilization (32) or mitochondrial protein aggregates (33). Growing evidence has highlighted that oxidative stress, characterized by overproduction of ROS, is also largely involved in mitochondrial disorders. Overproduction of ROS induces mitochondrial DNA mutation, ETC damage, membrane permeability and Ca^2+^ homeostasis alteration (34). Current treatment strategies investigate gene therapy of mitochondrial DNA (mtDNA) (35) or stem cell transplantation (36). In this context, MSCs have been reported to regulate oxidative stress and redox imbalance and may be of therapeutic interest to counteract mitochondrial disorders (28, 37). In addition, the energetic metabolism of MSCs regulate their immunomodulatory function and consequently their therapeutic properties (38).

## Mesenchymal Stromal Cells and Mitochondrial Metabolism of Target Cells

The exact role of MSCs on the mitochondrial metabolism of target cell is still unclear, but co-culture experiments clearly demonstrated the antioxidant properties of MSCs (28). MSCs act directly by reducing the oxidative stress (39) related to injury or inflammation, or indirectly by upregulating the antioxidant defences of target cells and altering cellular bioenergetics (37).

### Mesenchymal Stromal Cells Improve the Mitochondrial Function

The MSC potential to attenuate oxidative injury is based on the dampening of ROS production and enhancement of mitochondrial function (37). Reduction of ROS results from the secretion of anti-oxidant components [superoxyde dismutase (SOD), catalase, Glutathione S-transferases (GST), …], which re-equilibrate the redox balance in the host cell (40).

### Mesenchymal Stromal Cells Equilibrate Mitochondrial Dynamics

Both fission and fusion processes are linked to mitochondrial metabolism (28). Excessive mitochondrial fission is associated with reduction in OXPHOS and the balance between fusion and fission events is required to maintain cell homeostasis (41–44). Mitochondrial fusion is not essential for cell survival, but for normal development. MSCs can rescue aberrant morphology from a fission- to a fusion-like state and restore OXPHOS, by 962 increasing metabolic capacities and ROS production (28). They can induce expression of mitochondrial fusion genes [mitofusin (*mfn1*, *mfn2)* and optic atrophy 1 (Opa1)] protecting cells against environmental damages by improving respiration parameters (45).

### Mesenchymal Stromal Cells Minimize Mitochondrial Injury

MSCs can protect injured cells from mitochondria-related apoptosis and resulting oxidative damage by reducing release of CYTC into the cytoplasm. They also secrete a series of cytokines and growth factors, which upregulate anti-apoptotic proteins (BCL-XL, BCL-2), downregulate pro-apoptotic proteins (BAX, BAK, BAD) and CYTC and finally minimize mitochondria injury (2).

### Mesenchymal Stromal Cells Accelerate Mitochondria Recovery

Mitochondrial recovery is a strategy for restricting mitochondrial dysfunction and Sirtuin 3 (SIRT3) plays an important role in this process. Through SIRT3 activation and upregulation of peroxisome proliferator-activated receptor gamma coactivator 1-alpha (PGC-1α), mitochondrial biogenesis increases and ROS production decreases (46, 47). MSCs can enhance PGC-1α expression and normalise mitochondrial shape, density and mass (46), thereby re-equilibrating the cell energetic metabolism.

### Mesenchymal Stromal Cells Transfer Mitochondrial Cargo

Mitochondrial transfer mechanisms between MSCs and recipient cells are based on cell fusion, EV secretion, gap junctions or tunnelling nanotubes (48). In the acute phase of lung injury, internalized mitochondria were shown to increase ATP concentration in recipient cells, leading to bioenergetics restoration and cell protection (49). MSC mitochondrial transfer plays multiple roles, including tissue repair during injuries, apoptosis prevention in endothelial cells during ischemic stress or metabolic reprogramming (50, 51). Horizontal transfer of mitochondria or mitochondrial DNA between cells address therapeutic applications in MSCs regenerative medicine (48).

## Role of miRNAs in Mitochondrial Metabolism

### miRNAs: Synthesis, Mechanism, and Function

**Figure 2:**
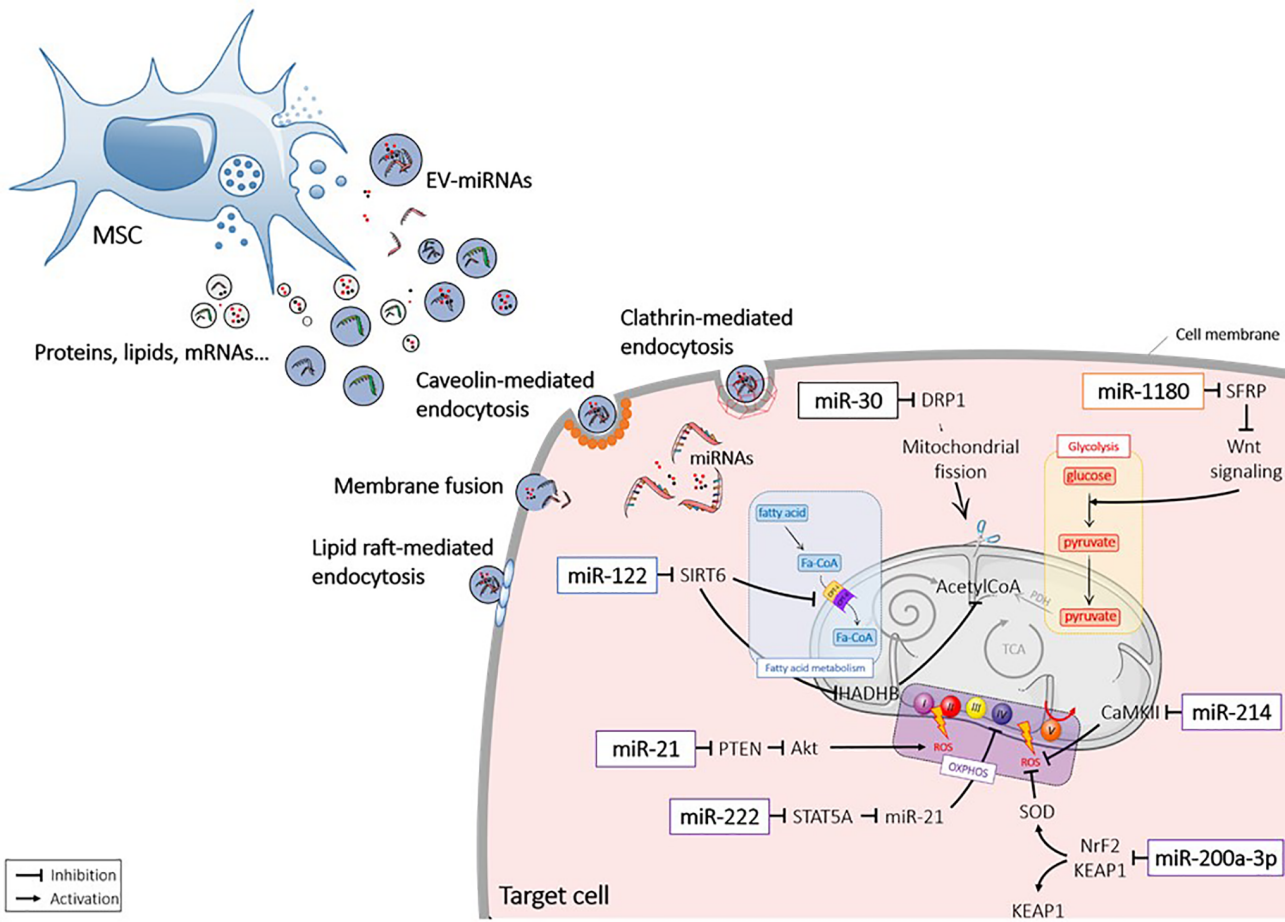
Extracellular vesicle-mediated delivery of miRNAs into recipient cells. Mesenchymal stromal cell (MSC) released extracellular vesicles (EVs) that contain miRNAs (EV-miRNAs). MiRNAs are taken up by the recipient cells *via* different mechanisms and act in the cytosol by interacting with targets. Akt1, protein kinase B; CamKII, calmodulin-dependent protein kinase II; DRP1, Dynamin-related protein 1; Fa-CoA. Fatty acid coenzyme A; HADHB, Hydroxyacyl-CoA Dehydrogenase Trifunctional Multienzyme Complex Subunit Beta; KEAP1, Kelch-like ECH-associated protein 1; Nrf2, NF-E2 p45-related factor 2; OXPHOS, oxidative phosphorylation; PDH, Pyruvate dehydrogenase; PTEN, Phosphatase and tensin homolog; ROS, reactive oxygen species; SFRP, secreted frizzled-related protein; SIRT, sirtuin; SOD, superoxydes dismutase; STAT, signal transducer and activator of transcription; TCA, tricarboxylic acid cycle; Wnt, wingless.

MicroRNAs (miRNAs) are non-coding endogenous ˜22nt (18 – 25nt) RNAs, which form a large family of post-transcriptional regulators. Within the nucleus, miRNA genes are transcribed by RNA polymerases as long precursor pri-miRNAs (> 500 bases), which harbour stem-loop structures (52). Maturation of miRNAs starts by the nuclear cleavage of the pri-miRNA by the Drosha RNA III endonuclease, which liberates a smaller pre-miRNA molecule. Pre-miRNAs are then exported to the cytoplasm by a GTP-dependent exportin (53). Once in the cytoplasm, pre-miRNAs are cleaved by an enzyme of the DICER family to release a small double-stranded RNA called “miRNA/miRNA duplex”. The passenger strand is generally degraded. The miRNA major strand binds to its mRNA target, which contains a perfectly complementary seed sequence on its 3’ UTR end (52, 54). MiRNAs act by post-transcriptional gene silencing or mRNA degradation and are involved in major biological processes, including cell metabolism (54–57). The process of miRNA and pre-miRNA sorting in EVs remains elusive (58). MiRNAs can diffuse through intracellular space to the plasma membrane and be loaded by the Annexin A2‐dependent pathway (59, 60). MiRNAs can also be transported by RNA-binding proteins (RBPs) toward multivesicular bodies (MVBs) and be packed by budding-in process (61) [for review see (62)]. Several studies indicated that miRNA packaging in EVs could serve as a major mechanism of miRNA transfer between cells. Of interest, there is increasing evidence for the antioxidant role of miRNAs contained within MSC-EVs for different therapeutic applications (12, 63) (**Figure 2**).

### miRNAs Target Mitochondrial Metabolic Pathways

Recent studies have shown that miRNAs are able to translocate into the mitochondrial compartment and modulate mitochondrial activities (30). A particular miRNA subset, called mitomiR for mitochondrial microRNAs, is localized within mitochondria and transcribed either from nuclear or mitochondrial genome (64). Imported mature mitomiRs are translocated after pre-miRNAs are processed by DICER (65). Among mitomiRs, some have been described as specific regulators of mitochondrial metabolism (66). As an example, mir-149 targets Poly(ADP-Ribose) Polymerase-2 (PARP-2) that activates SIRT1 and subsequently, increases mitochondrial function and biogenesis *via* PGC-1 activation (66). MiRNAs target directly or indirectly a number of key enzymes involved in glycolysis regulation (67). One such example is miR-326 that is overexpressed in metabolically active cancer cells and targets Pyruvate Kinase M2 (PKM2) (68). Some miRNAs are involved in the repair of damages induced by ROS (69). MiR-128a targets Polycomb complex protein (BMI-1) involved in the mitochondrial and redox homeostasis and cellular senescence in a medulloblastoma model (70). In absence of BMI-1, ETC flow is interrupted decreasing ROS generation. Other miRNAs, such as miR-25, target genes that play crucial roles in Ca^2+^ uptake and consequently, ROS production by targeting Mitochondrial Calcium Uniporter (MCU) (71).

### miRNAs and Oxidative Phosphorylation

MiRNAs can target OXPHOS either directly, by targeting mRNAs of essential ETC factors and/or indirectly, by targeting the biosynthesis pathways of essential cofactors (17). MiR-338 and miR-181c have been shown to target complex IV of respiratory chain (COXIV) (21, 72). Overexpression of miR-338 decreased COXIV levels and subsequently, oxidative phosphorylation while miR-181c remodels COXIV by targeting cytochrome c oxidase subunit 1 (COX1) mRNA (72, 73). ATP synthase is a direct target of miR-101 and miR-127 (74). MiR-210 affects the cytoplasmic Iron Sulphur Cluster homologue (ISCU) and blocks ETC (21). SIRT4, which modulates mitochondrial oxidative capacity by targeting glutamate dehydrogenase (GDH), is regulated by miR-15b (75). Inhibition of miR-15b promotes mitochondrial ROS and decreases mitochondrial membrane potential in a SIRT4-dependent manner (76).

### miRNAs and TriCarboxylic Acid Cycle

MiRNAs can target central biochemical reactions, shared by many pathways. For example, the citrate synthase from the TCA cycle is targeted by a set of miRNAs: miR-152, miR-148a, miR-148b, miR-299-5p, miR-19b, miR-122a, miR-421, miR-494, and miR-19a (77). As a result, 78 pathways including the purine metabolism, pentose phosphate pathway, fatty acid biosynthesis, as well as carbon, nucleotide and amino acid metabolisms are affected (78). Another enzyme of TCA cycle, Aconitate hydratase (ACO2), which metabolizes α-ketoglutarate, a product of glutamine oxidation is targeted by miR-450 (79). MiR-450 decreases mitochondrial membrane potential and increases glucose uptake (80).

### miRNAs and Amino Acid Metabolism

Many miRNAs, including miR-23a/b (81), miR-137 (82), miR-153 (83) and miR192/204 (84), target glutaminase and modulate ROS production. Another enzyme, Serine Hydroxyl-Methyl Transferase (SHMT), which converts serine to glycine, is targeted by several miRNAs, such as miR-193 and miR-642a-5p (85, 86). The Phosphoserine aminotransferase (PSAT1) which catalyzes the reversible conversion of 3-phosphohydroxypyruvate (3-PHP) to phosphoserine (3-PS) is also targeted by miR-340 (87) and miR-29a-3p (Bony et al., submitted).

### miRNAs and Fatty Acid Metabolism

Several miRNAs have been described to regulate lipid metabolism. MiR-370, miR-148a, and miR-33 target CPT1A, which is responsible for the translocation of fatty acids from the cytosol to mitochondrial matrix, thereby reducing fatty acid oxidation (88–90). MiR-696 regulates fatty acid oxidation by targeting PGC-1α (91).

The regulation of mitochondrial proteins by miRNAs can therefore modulate mitochondrial function suggesting that targeting miRNAs might provide a new therapeutic approach for the treatment of mitochondria-related diseases.

## Role of Extracellular Vesicles-miRNAs on Mitochondrial Metabolism Regulation in Diseases

### Role of Mesenchymal Stromal Cell-Derived Extracellular Vesicles in the Regulation of Mitochondrial Metabolism

Various studies have shown that EVs from different cell types can release miRNAs in the cytoplasm of recipient cells where they target mitochondrial metabolism pathways (92). Exposure to EVs significantly increases the mitochondrial respiration, especially through the increase of ROS production and ATP production (93, 94). Notably, MSC-derived EVs (MSC-EVs) play a central role in promoting mitochondrial function. Proteomic and RNAseq analysis demonstrated that MSC-EVs contain several proteins, genes, mRNA, and miRNA involved in glycolysis such as GAPDH, Glucose-6-phosphate isomerase (GPI), in the TCA cycle [2-oxoglutarate dehydrogenase (OGDH)], and ETC (ATPase). In the rat model of pulmonary arterial hypertension (PAH), MSC-EVs upregulated both PDH and Glutamate Dehydrogenase 1 (GLUD1) gene expression in an hypoxic environment and increased glucose oxidation by TCA cycle (92). In a model of ischemia-reperfusion injury, umbilical cord MSC-EVs were shown to contain manganese-dependent superoxide dismutase (MnSOD), which decreased ROS levels and prevented oxidative stress in hepatic tissue (95).

### MiRNAs Loaded by Mesenchymal Stromal Cell-Extracellular Vesicles Improve Mitochondrial Dysfunction in Pathological Conditions

#### Renal Diseases

Acute kidney injury (AKI) results from severe renal ischemia reperfusion injury (IRI), a common clinical situation during transplantation. Renal reperfusion induces oxidative stress, characterized by increased ROS and/or reactive nitrogen species (RNS) (96). MSC-EVs have been shown to alleviate AKI through miRNA transfer to resident renal cells (97).

##### miR-30

In a rat model of AKI, IRI caused a lower expression of miR-30-b, miR-30-c, and miR-30-d in renal cells. MSC-EVs were shown to protect kidney from IRI by inhibiting mitochondrial fission through miR-30 transfer (98). Transfer of miR-30 mediated the regenerative effect of MSCs by decreasing Dynamin-related protein 1 (DRP1), which induces mitochondrial fragmentation, and subsequently protecting kidney from ischemia (63).

##### miR-200a-3p

MSC-EVs have been reported to stimulate ATP production and the antioxidant defense of tubular epithelia cells (TECs) through activation of KEAP1-NRF2 signaling pathway (99). *In vivo*, MSC-EVs were shown to significantly increase the expression of the antioxidant NRF2 and SOD2, both at the protein and mRNA level, while the pro-oxidant KEAP1 was decreased. By investigating the miRNA profile of injured kidney cells, miR-200a-3p was significantly increased after MSC-EV injection. Importantly, EVs isolated from antagomiR-200a-3p-treated MSCs failed to preserve *in vitro* the structural integrity of mitochondria in TECs under oxidative damage. The authors concluded that miR-200a-3p was shuttled by MSC-EVs to target the KEAP1-NRF2 signaling pathway.

##### miR-222

In diabetes, hyperglycemia (HG) leads to the down-regulation of COXIV in the ETC complex, leading to mitochondrial disorders and renal tissue alterations. Transfer of miR-222 from MSC-EVs to mesangial cells reduced STAT5A expression, leading to miR-21 down-regulation (100). A previous study showed that miR-21 silencing enhanced mitochondrial function, by reducing ROS production (101). Therefore, MSC-EVs rescued COXIV expression in mesangial cells by miR-222 transfer and miR-21 down-regulation.

#### Cardiac Diseases

Cardiac stem cells (CSCs) have been reported to be largely involved in cardiac regeneration and repair after injury [for review, see (90)]. CSCs have emerged as a promising therapeutic tool but their poor survival and engraftment prevent their use. However, endogenous CSC function can be improved by miRNAs released by MSCs-EVs after heart damage (102–104).

##### miR-214

Previous studies indicated that miR-214 was up-regulated by hypoxic stress to protect cardiac myocytes from damage (105). In a hypoxic context, MSC-EVs were reported to decrease the apoptotic rate of CSCs and the production of ROS mediated by CaMKII activation, which is a direct target of miR-214 (106). Transfer of miR-214 by MSC-EVs is likely one of the main effector that protects CSCs from oxidative damage in myocardial infarction.

##### miR-21

Mir-21 is another miRNA whose cardioprotective role has been demonstrated (107). To mimic the pathophysiology of cardiovascular environment, MSC-EVs were collected from H_2_O_2_-treated MSCs and showed to contain higher levels of miR-21 than EVs from non-treated MSCs. Actually, miR-21 up-regulation in H_2_o_2_-treated MSC-EVs was shown to regulate the apoptosis of CSCs through PTEN down-regulation and PI3K/AKT activation.

#### Ovarian Cancer

In the 1920s, Otto Warburg described that cancer cells use higher levels of glucose than normal cells, thereby leading to increased lactate production (108). Since, this phenomenon of aerobic glycolysis, called Warburg’s effect, has been observed in different tumours, including, breast, lung, colorectal cancer and glioblastoma (109) and the role of MSC-EVs in regulating tumour metabolism has been investigated.

##### miR-1180

MiR-1180 was the most abundant miRNA detected in the conditioned medium of MSCs that were able to stimulate both glycolysis of ovarian cancer cells and chemoresistance to cisplatin treatment (110, 111). Overexpression of miR-1180 in MSCs resulted in the activation of Wnt signaling pathway and expression of its downstream components (Wnt5a, β-catenin, c-Myc…) responsible for glycolysis-induced chemoresistance. Conversely, the supernatant of MSCs treated with miR-1180 inhibitors suppressed cell proliferation and ATP production in cancer cells and restored their chemosensitivity. The effect of MSC-EVs was associated with the down-regulation of Secreted Frizzled-Related Protein 1 (SFRP1), a negative regulator of the Wnt signaling pathway, by miR-1180. Thus, MSC-derived miR-1180 stimulated cancer cell proliferation by stimulating glycolysis-induced chemoresistance.

#### Liver Diseases

Previous studies have demonstrated that MSCs can reduce inflammatory responses, hepatocyte apoptosis, liver fibrosis and, enhance liver regeneration and functionality [for review, see (101)]. This therapeutic effect of MSCs was attributed to their capacities to secrete trophic factors (111, 112).

##### miR-122

MiR-122 has a critical role in liver function as being involved in cholesterol biosynthesis, fatty acid synthesis and β-oxidation (113). In normal conditions, miR-122 is one of the most abundant miRNAs, but its expression is reduced in advanced liver diseases. MSC-EVs from patients cannot prevent liver impairment but miR-122-engineered MSCs can release EVs containing high amounts of miR-122. Hepatic cells incubated with miR-122-containing EVs expressed lower levels of miR-122 target genes, including Prolyl 4-hydroxylase subunit alpha-1 (P4HA1) involved in collagen synthesis and insulin-like growth factor 1 receptor (IGF1R) (114). Mir-122 also repressed SIRT6 leading to lower expression levels of acetyl-CoA acyltransferase (HADHB) and carnitine palmitoyltransferase I (CPT1), involved in fatty acid synthesis and β-oxidation (115). MiR-122 is therefore proposed as a key regulator of liver function playing a central role in lipid metabolism and the regulation of metabolic diseases.

## Conclusion

In the current study, we described various miRNAs that are expressed by MSCs and packaged in EVs and that directly or indirectly regulate critical functions of mitochondria. MSC-derived miRNAs are promising actors in regenerative medicine and in therapeutics as they can modulate several pathways that are altered in mitochondrial dysfunction-related diseases. Data are still sparse but encouraging results have been reported. Manipulating miRNA expression using miRNA mimics and inhibitors may serve as potential therapeutic approach for diverse diseases. A better understanding of miRNA biogenesis, import and function is needed but should provide new insights on the feasibility of this novel strategy for mitochondria-associated disorders.

## Author Contributions

All authors contributed to the article and approved the submitted version.

## Funding

We gratefully acknowledge the Agence Nationale pour la Recherche for support of the national infrastructure: “ECELLFRANCE: Development of a national adult mesenchymal stem cell based therapy platform” (ANR-11-INSB-005). The study was also supported by the European Union Horizon 2020 Programme (project RESPINE, grant agreement #: 732163). The materials presented and views expressed here are the responsibility of the authors only. The EU Commission takes no responsibility for any use made of the information set out. Funding for staff exchange was received by Programme Ulysses 2018 (project #: P41059WK).

## Conflict of Interest

The authors declare that the research was conducted in the absence of any commercial or financial relationships that could be construed as a potential conflict of interest.

## References

[1] Duarte FV Amorim JA Palmeira CM Rolo AP. Regulation of Mitochondrial Function and its Impact in Metabolic Stress. Curr Med Chem (2015) 22:2468–79. 10.2174/0929867322666150514095910 25973983

[2] Zhao L Hu C Zhang P Jiang H Chen J. Mesenchymal stem cell therapy targeting mitochondrial dysfunction in acute kidney injury. J Transl Med (2019) 17:142. 10.1186/s12967-019-1893-4 31046805 PMC6498508

[3] Friedenstein AJ Gorskaja JF Kulagina NN. Fibroblast precursors in normal and irradiated mouse hematopoietic organs. Exp Hematol (1976) 4:267–74. 976387

[4] da Silva Meirelles L Chagastelles PC Nardi NB. Mesenchymal stem cells reside in virtually all post-natal organs and tissues. J Cell Sci (2006) 119:2204–13. 10.1242/jcs.02932 16684817

[5] Viswanathan S Shi Y Galipeau J Krampera M Leblanc K Martin I. Mesenchymal stem versus stromal cells: International Society for Cell & Gene Therapy (ISCT®) Mesenchymal Stromal Cell committee position statement on nomenclature. Cytotherapy (2019) 21:1019–24. 10.1016/j.jcyt.2019.08.002 31526643

[6] Moll G Ankrum JA Kamhieh-Milz J Bieback K Ringdén O Volk H-D. Intravascular Mesenchymal Stromal/Stem Cell Therapy Product Diversification: Time for New Clinical Guidelines. Trends Mol Med (2019) 25:149–63. 10.1016/j.molmed.2018.12.006 30711482

[7] Maumus M Jorgensen C Noël D. Mesenchymal stem cells in regenerative medicine applied to rheumatic diseases: Role of secretome and exosomes. Biochimie (2013) 95:2229–34. 10.1016/j.biochi.2013.04.017 23685070

[8] Keyser KA Beagles KE Kiem H-P. Comparison of mesenchymal stem cells from different tissues to suppress T-cell activation. Cell Transplant (2007) 16:555–62. 10.3727/000000007783464939 17708345

[9] Kern S Eichler H Stoeve J Klüter H Bieback K. Comparative Analysis of Mesenchymal Stem Cells from Bone Marrow, Umbilical Cord Blood, or Adipose Tissue. Stem Cells (2006) 24:1294–301. 10.1634/stemcells.2005-0342 16410387

[10] Luz-Crawford P Djouad F Toupet K Bony C Franquesa M Hoogduijn MJ. Mesenchymal Stem Cell-Derived Interleukin 1 Receptor Antagonist Promotes Macrophage Polarization and Inhibits B Cell Differentiation. Stem Cells (2016) 34:483–92. 10.1002/stem.2254 26661518

[11] Doorn J Moll G Le Blanc K van Blitterswijk C de Boer J. Therapeutic applications of mesenchymal stromal cells: paracrine effects and potential improvements. Tissue Eng Part B Rev (2012) 18:101–15. 10.1089/ten.TEB.2011.0488 21995703

[12] Park K-S Bandeira E Shelke GV Lässer C Lötvall J. Enhancement of therapeutic potential of mesenchymal stem cell-derived extracellular vesicles. Stem Cell Res Ther (2019) 10:288. 10.1186/s13287-019-1398-3 31547882 PMC6757418

[13] Akers JC Gonda D Kim R Carter BS Chen CC. Biogenesis of extracellular vesicles (EV): exosomes, microvesicles, retrovirus-like vesicles, and apoptotic bodies. J Neurooncol (2013) 113:1–11. 10.1007/s11060-013-1084-8 23456661 PMC5533094

[14] Abels ER Breakefield XO. Introduction to Extracellular Vesicles: Biogenesis, RNA Cargo Selection, Content, Release, and Uptake. Cell Mol Neurobiol (2016) 36:301–12. 10.1007/s10571-016-0366-z PMC5546313 27053351

[15] Colombo M Raposo G Théry C. Biogenesis, secretion, and intercellular interactions of exosomes and other extracellular vesicles. Annu Rev Cell Dev Biol (2014) 30:255–89. 10.1146/annurev-cellbio-101512-122326 25288114

[16] Asgarpour K Shojaei Z Amiri F Ai J Mahjoubin-Tehran M Ghasemi F. Exosomal microRNAs derived from mesenchymal stem cells: cell-to-cell messages. Cell Commun Signal (2020) 18:149. 10.1186/s12964-020-00650-6 32917227 PMC7488404

[17] Geiger J Dalgaard LT. Interplay of mitochondrial metabolism and microRNAs. Cell Mol Life Sci (2017) 74:631–46. 10.1007/s00018-016-2342-7 PMC11107739 27563705

[18] Osellame LD Blacker TS Duchen MR. Cellular and molecular mechanisms of mitochondrial function. Best Pract Res Clin Endocrinol Metab (2012) 26:711–23. 10.1016/j.beem.2012.05.003 PMC3513836 23168274

[19] Prasai K. Regulation of mitochondrial structure and function by protein import: A current review. Pathophysiology (2017) 24:107–22. 10.1016/j.pathophys.2017.03.001 28400074

[20] McBride HM Neuspiel M Wasiak S. Mitochondria: More Than Just a Powerhouse. Curr Biol (2006) 16:R551–60. 10.1016/j.cub.2006.06.054 16860735

[21] Li P Jiao J Gao G Prabhakar BS. Control of mitochondrial activity by miRNAs. J Cell Biochem (2012) 113:1104–10. 10.1002/jcb.24004 PMC3325319 22135235

[22] Orrenius S. Reactive Oxygen Species in Mitochondria-Mediated Cell Death. Drug Metab Rev (2007) 39:443–55. 10.1080/03602530701468516 17786631

[23] Dröge W. Free radicals in the physiological control of cell function. Physiol Rev (2002) 82:47–95. 10.1152/physrev.00018.2001 11773609

[24] Qutub AA Popel AS. Reactive oxygen species regulate hypoxia-inducible factor 1alpha differentially in cancer and ischemia. Mol Cell Biol (2008) 28:5106–19. 10.1128/MCB.00060-08 PMC2519710 18559422

[25] Vidali S Aminzadeh S Lambert B Rutherford T Sperl W Kofler B. Mitochondria: The ketogenic diet–A metabolism-based therapy. Int J Biochem Cell Biol (2015) 63:55–9. 10.1016/j.biocel.2015.01.022 25666556

[26] Munday MR. Regulation of mammalian acetyl-CoA carboxylase. Biochem Soc Trans (2002) 30:1059–64. 10.1042/bst0301059 12440972

[27] van der Bliek AM Sedensky MM Morgan PG. Cell Biology of the Mitochondrion. Genetics (2017) 207:843–71. 10.1534/genetics.117.300262 PMC5676242 29097398

[28] Newell C Sabouny R Hittel Dustin S Shutt TE Khan A Klein MS. Mesenchymal Stem Cells Shift Mitochondrial Dynamics and Enhance Oxidative Phosphorylation in Recipient Cells. Front Physiol (2018) 9:1–6. 10.3389/fphys.2018.01572 30555336 PMC6282049

[29] Pieczenik SR Neustadt J. Mitochondrial dysfunction and molecular pathways of disease. Exp Mol Pathol (2007) 83:84–92. 10.1016/j.yexmp.2006.09.008 17239370

[30] Tuppen HAL Blakely EL Turnbull DM Taylor RW. Mitochondrial DNA mutations and human disease. Biochim Biophys Acta (2010) 1797:113–28. 10.1016/j.bbabio.2009.09.005 19761752

[31] Vannuvel K Renard P Raes M Arnould T. Functional and morphological impact of ER stress on mitochondria. J Cell Physiol (2013) 228:1802–18. 10.1002/jcp.24360 23629871

[32] Dudkina NV Kouril R Peters K Braun H-P Boekema EJ. Structure and function of mitochondrial supercomplexes. Biochim Biophys Acta (2010) 1797:664–70. 10.1016/j.bbabio.2009.12.013 20036212

[33] Pellegrino MW Nargund AM Haynes CM. Signaling the mitochondrial unfolded protein response. Biochim Biophys Acta (2013) 1833:410–6. 10.1016/j.bbamcr.2012.02.019 PMC3393825 22445420

[34] Stepien KM Roncaroli F Turton N Hendriksz CJ Roberts M Heaton RA. Mechanisms of Mitochondrial Dysfunction in Lysosomal Storage Disorders: A Review. J Clin Med (2020) 9:1–22. 10.3390/jcm9082596 PMC7463786 32796538

[35] Craven L Alston CL Taylor RW Turnbull DM. Recent Advances in Mitochondrial Disease. Annu Rev Genomics Hum Genet (2017) 18:257–75. 10.1146/annurev-genom-091416-035426 28415858

[36] Wallace DC Fan W Procaccio V. Mitochondrial energetics and therapeutics. Annu Rev Pathol (2010) 5:297–348. 10.1146/annurev.pathol.4.110807.092314 20078222 PMC3245719

[37] Stavely R Nurgali K. The emerging antioxidant paradigm of mesenchymal stem cell therapy. Stem Cells Transl Med (2020) 9:985–1006. 10.1002/sctm.19-0446 32497410 PMC7445024

[38] Yuan X Logan TM Ma T. Metabolism in Human Mesenchymal Stromal Cells: A Missing Link Between hMSC Biomanufacturing and Therapy? Front Immunol (2019) 10:1–11. 10.3389/fimmu.2019.00977 31139179 PMC6518338

[39] Denu RA Hematti P. Effects of Oxidative Stress on Mesenchymal Stem Cell Biology. Oxid Med Cell Longev (2016) 2016:2989076. 10.1155/2016/2989076 27413419 PMC4928004

[40] Redondo J Sarkar P Kemp K Heesom KJ Wilkins A Scolding NJ. Dysregulation of Mesenchymal Stromal Cell Antioxidant Responses in Progressive Multiple Sclerosis. Stem Cells Transl Med (2018) 7:748–58. 10.1002/sctm.18-0045 PMC6186266 30063300

[41] Sabouny R Shutt TE. Reciprocal Regulation of Mitochondrial Fission and Fusion. Trends Biochem Sci (2020) 45:564–77. 10.1016/j.tibs.2020.03.009 32291139

[42] Sebastián D Palacín M Zorzano A. Mitochondrial Dynamics: Coupling Mitochondrial Fitness with Healthy Aging. Trends Mol Med (2017) 23:201–15. 10.1016/j.molmed.2017.01.003 28188102

[43] Wai T Langer T. Mitochondrial Dynamics and Metabolic Regulation. Trends Endocrinol Metab (2016) 27:105–17. 10.1016/j.tem.2015.12.001 26754340

[44] Samudio I Fiegl M McQueen T Clise-Dwyer K Andreeff M. The Warburg effect in leukemia-stroma cocultures is mediated by mitochondrial uncoupling associated with uncoupling protein 2 activation. Cancer Res (2008) 68:5198–205. 10.1158/0008-5472.CAN-08-0555 PMC2562568 18593920

[45] Maremanda KP Sundar IK Rahman I. Protective role of mesenchymal stem cells and mesenchymal stem cell-derived exosomes in cigarette smoke-induced mitochondrial dysfunction in mice. Toxicol Appl Pharmacol (2019) 385:114788. 10.1016/j.taap.2019.114788 31678243 PMC6894395

[46] Perico L Morigi M Rota C Breno M Mele C Noris M. Human mesenchymal stromal cells transplanted into mice stimulate renal tubular cells and enhance mitochondrial function. Nat Commun (2017) 8:983. 10.1038/s41467-017-00937-2 29042548 PMC5754365

[47] Bell EL Guarente L. The SirT3 divining rod points to oxidative stress. Mol Cell (2011) 42:561–8. 10.1016/j.molcel.2011.05.008 PMC3526939 21658599

[48] Mohammadalipour A Dumbali SP Wenzel PL. Mitochondrial Transfer and Regulators of Mesenchymal Stromal Cell Function and Therapeutic Efficacy. Front Cell Dev Biol (2020) 8:603292. 10.3389/fcell.2020.603292 33365311 PMC7750467

[49] Liang X Ding Y Zhang Y Tse H-F Lian Q. Paracrine mechanisms of mesenchymal stem cell-based therapy: current status and perspectives. Cell Transplant (2014) 23:1045–59. 10.3727/096368913X667709 23676629

[50] Spees JL Lee RH Gregory CA. Mechanisms of mesenchymal stem/stromal cell function. Stem Cell Res Ther (2016) 7:125. 10.1186/s13287-016-0363-7 27581859 PMC5007684

[51] Acquistapace A Bru T Lesault P-F Figeac F Coudert AE le Coz O. Human mesenchymal stem cells reprogram adult cardiomyocytes toward a progenitor-like state through partial cell fusion and mitochondria transfer. Stem Cells (2011) 29:812–24. 10.1002/stem.632 PMC3346716 21433223

[52] Lee Y Kim M Han J Yeom KH Lee S Baek SH. MicroRNA genes are transcribed by RNA polymerase II. EMBO J (2004) 23:4051–60. 10.1038/sj.emboj.7600385 PMC524334 15372072

[53] Bohnsack MT Czaplinski K Gorlich D. Exportin 5 is a RanGTP-dependent dsRNA-binding protein that mediates nuclear export of pre-miRNAs. Rna (2004) 10:185–91. 10.1261/rna.5167604 PMC1370530 14730017

[54] Bartel DP. MicroRNAs: genomics, biogenesis, mechanism, and function. Cell (2004) 116:281–97. 10.1016/S0092-8674(04)00045-5 14744438

[55] O’Brien J Hayder H Zayed Y Peng C. Overview of MicroRNA Biogenesis, Mechanisms of Actions, and Circulation. Front Endocrinol (2018) 9:1–12. 10.3389/fendo.2018.00402 PMC6085463 30123182

[56] Rottiers V Näär AM. MicroRNAs in metabolism and metabolic disorders. Nat Rev Mol Cell Biol (2012) 13:239–50. 10.1038/nrm3313 PMC4021399 22436747

[57] Wahid F Shehzad A Khan T Kim YY. MicroRNAs: Synthesis, mechanism, function, and recent clinical trials. Biochim Biophys Acta (BBA) Mol Cell Res (2010) 1803:1231–43. 10.1016/j.bbamcr.2010.06.013 20619301

[58] Munir J Yoon JK Ryu S. Therapeutic miRNA-Enriched Extracellular Vesicles: Current Approaches and Future Prospects. Cells (2020) 9:1–18. 10.3390/cells9102271 PMC7601381 33050562

[59] Mills J Capece M Cocucci E Tessari A Palmieri D. Cancer-Derived Extracellular Vesicle-Associated MicroRNAs in Intercellular Communication: One Cell’s Trash Is Another Cell’s Treasure. Int J Mol Sci (2019) 20:1–27. 10.3390/ijms20246109 PMC6940802 31817101

[60] Hagiwara K Katsuda T Gailhouste L Kosaka N Ochiya T. Commitment of Annexin A2 in recruitment of microRNAs into extracellular vesicles. FEBS Lett (2015) 589:4071–8. 10.1016/j.febslet.2015.11.036 26632510

[61] Chen J Hu C Pan P. Extracellular Vesicle MicroRNA Transfer in Lung Diseases. Front Physiol (2017) 8:1–9. 10.3389/fphys.2017.01028 29311962 PMC5732924

[62] Janas T Janas MM Sapoń K Janas T. Mechanisms of RNA loading into exosomes. FEBS Lett (2015) 589:1391–8. 10.1016/j.febslet.2015.04.036 25937124

[63] Qiu G Zheng G Ge M Wang J Huang R Shu Q. Mesenchymal stem cell-derived extracellular vesicles affect disease outcomes via transfer of microRNAs. Stem Cell Res Ther (2018) 9:1–9. 10.1186/s13287-018-1069-9 30463593 PMC6249826

[64] Bandiera S Matégot R Girard M Demongeot J Henrion-Caude A. MitomiRs delineating the intracellular localization of microRNAs at mitochondria. Free Radic Biol Med (2013) 64:12–9. 10.1016/j.freeradbiomed.2013.06.013 23792138

[65] Song R Hu X-Q Zhang L. Mitochondrial MiRNA in Cardiovascular Function and Disease. Cells (2019) 8:1–13. 10.3390/cells8121475 PMC6952824 31766319

[66] Duarte FV Palmeira CM Rolo AP. The Role of microRNAs in Mitochondria: Small Players Acting Wide. Genes (Basel) (2014) 5:865–86. 10.3390/genes5040865 PMC4276918 25264560

[67] Singh PK Mehla K Hollingsworth MA Johnson KR. Regulation of Aerobic Glycolysis by microRNAs in Cancer. Mol Cell Pharmacol (2011) 3:125–34. PMC3392682 22792411

[68] Kefas B Comeau L Erdle N Montgomery E Amos S Purow B. Pyruvate kinase M2 is a target of the tumor-suppressive microRNA-326 and regulates the survival of glioma cells. Neuro-oncology (2010) 12:1102–12. 10.1093/neuonc/noq080 PMC3098027 20667897

[69] Torma F Gombos Z Jokai M Berkes I Takeda M Mimura T. The roles of microRNA in redox metabolism and exercise-mediated adaptation. J Sport Health Sci (2020) 9:405–14. 10.1016/j.jshs.2020.03.004 PMC7498669 32780693

[70] Babu K R Tay Y. The Yin-Yang Regulation of Reactive Oxygen Species and MicroRNAs in Cancer. Int J Mol Sci (2019) 20:1–21. 10.3390/ijms20215335 PMC6862169 31717786

[71] Jaquenod De Giusti C Roman B Das S. The Influence of MicroRNAs on Mitochondrial Calcium. Front Physiol (2018) 9:1291. 10.3389/fphys.2018.01291 30298016 PMC6160583

[72] Das S Ferlito M Kent OA Fox-Talbot K Wang R Liu D. Nuclear miRNA regulates the mitochondrial genome in the heart. Circ Res (2012) 110:1596–603. 10.1161/CIRCRESAHA.112.267732 PMC3390752 22518031

[73] Aschrafi A Kar AN Natera-Naranjo O MacGibeny MA Gioio AE Kaplan BB. MicroRNA-338 regulates the axonal expression of multiple nuclear-encoded mitochondrial mRNAs encoding subunits of the oxidative phosphorylation machinery. Cell Mol Life Sci (2012) 69:4017–27. 10.1007/s00018-012-1064-8 PMC11114659 22773120

[74] Baradan R Hollander JM Das S. Mitochondrial miRNAs in diabetes: just the tip of the iceberg. Can J Physiol Pharmacol (2017) 95:1156–62. 10.1139/cjpp-2016-0580 PMC5854153 28467860

[75] Lang A Grether-Beck S Singh M Kuck F Jakob S Kefalas A. MicroRNA-15b regulates mitochondrial ROS production and the senescence-associated secretory phenotype through sirtuin 4/SIRT4. Aging (Albany NY) (2016) 8:484–505. 10.18632/aging.100905 26959556 PMC4833141

[76] Haigis MC Mostoslavsky R Haigis KM Fahie K Christodoulou DC Murphy AJ. SIRT4 inhibits glutamate dehydrogenase and opposes the effects of calorie restriction in pancreatic beta cells. Cell (2006) 126:941–54. 10.1016/j.cell.2006.06.057 16959573

[77] Tomasetti M Amati M Santarelli L Neuzil J. MicroRNA in Metabolic Re-Programming and Their Role in Tumorigenesis. Int J Mol Sci (2016) 17:1–19. 10.3390/ijms17050754 PMC4881575 27213336

[78] Tibiche C Wang E. MicroRNA Regulatory Patterns on the Human Metabolic Network. Open Syst Biol J (2008) 1:1–8. 10.2174/1876392800801010001

[79] miR-450a Acts as a Tumor Suppressor in Ovarian Cancer by Regulating Energy Metabolism | Cancer Research. Available at: https://cancerres-aacrjournals-org.proxy.insermbiblio.inist.fr/content/79/13/3294.long (Accessed April 30, 2020). 10.1158/0008-5472.CAN-19-0490 PMC6606360 31101765

[80] Muys BR Sousa JF Plaça JR de Araújo LF Sarshad AA Anastasakis DG. miR-450a Acts as a Tumor Suppressor in Ovarian Cancer by Regulating Energy Metabolism. Cancer Res (2019) 79:3294–305. 10.1158/0008-5472.CAN-19-0490 PMC6606360 31101765

[81] Gao P Tchernyshyov I Chang T-C Lee Y-S Kita K Ochi T. c-Myc suppression of miR-23a/b enhances mitochondrial glutaminase expression and glutamine metabolism. Nature (2009) 458:762–5. 10.1038/nature07823 PMC2729443 19219026

[82] Luan W Zhou Z Zhu Y Xia Y Wang J Xu B. miR-137 inhibits glutamine catabolism and growth of malignant melanoma by targeting glutaminase. Biochem Biophys Res Commun (2018) 495:46–52. 10.1016/j.bbrc.2017.10.152 29097210

[83] Liu Z Wang J Li Y Fan J Chen L Xu R. MicroRNA-153 regulates glutamine metabolism in glioblastoma through targeting glutaminase. Tumour Biol (2017) 39:1010428317691429. 10.1177/1010428317691429 28218035

[84] Ge Y Yan X Jin Y Yang X Yu X Zhou L. MiRNA-192 [corrected] and miRNA-204 Directly Suppress lncRNA HOTTIP and Interrupt GLS1-Mediated Glutaminolysis in Hepatocellular Carcinoma. PloS Genet (2015) 11:e1005726. 10.1371/journal.pgen.1005726 26710269 PMC4692503

[85] Leivonen S-K Rokka A Östling P Kohonen P Corthals GL Kallioniemi O. Identification of miR-193b Targets in Breast Cancer Cells and Systems Biological Analysis of Their Functional Impact. Mol Cell Proteomics (2011) 10:1–9. 10.1074/mcp.M110.005322 PMC3134067 21512034

[86] Lin C Zhang Y Chen Y Bai Y Zhang Y. Long noncoding RNA LINC01234 promotes serine hydroxymethyltransferase 2 expression and proliferation by competitively binding miR-642a-5p in colon cancer. Cell Death Dis (2019) 10:1–16. 10.1038/s41419-019-1352-4 PMC6372696 30755591

[87] Yan S Jiang H Fang S Yin F Wang Z Jia Y. MicroRNA-340 Inhibits Esophageal Cancer Cell Growth and Invasion by Targeting Phosphoserine Aminotransferase 1. Cell Physiol Biochem (2015) 37:375–86. 10.1159/000430361 26316084

[88] Fernández-Hernando C Suárez Y Rayner KJ Moore KJ. MicroRNAs in lipid metabolism. Curr Opin Lipidol (2011) 22:86–92. 10.1097/MOL.0b013e3283428d9d 21178770 PMC3096067

[89] Karunakaran D Thrush AB Nguyen M-A Richards L Geoffrion M Singaravelu R. Macrophage Mitochondrial Energy Status Regulates Cholesterol Efflux and Is Enhanced by Anti-miR33 in Atherosclerosis. Circ Res (2015) 117:266–78. 10.1161/CIRCRESAHA.117.305624 PMC4578799 26002865

[90] Wagschal A Najafi-Shoushtari SH Wang L Goedeke L Sinha S deLemos AS. Genome-wide identification of microRNAs regulating cholesterol and triglyceride homeostasis. Nat Med (2015) 21:1290–7. 10.1038/nm.3980 PMC4993048 26501192

[91] Aoi W Naito Y Mizushima K Takanami Y Kawai Y Ichikawa H. The microRNA miR-696 regulates PGC-1{alpha} in mouse skeletal muscle in response to physical activity. Am J Physiol Endocrinol Metab (2010) 298:E799–806. 10.1152/ajpendo.00448.2009 20086200

[92] Hogan SE Rodriguez Salazar MP Cheadle J Glenn R Medrano C Petersen TH. Mesenchymal stromal cell-derived exosomes improve mitochondrial health in pulmonary arterial hypertension. Am J Physiol Lung Cell Mol Physiol (2019) 316:L723–37. 10.1152/ajplung.00058.2018 30652491

[93] Russell AE Jun S Sarkar S Geldenhuys WJ Lewis SE Rellick SL. Extracellular Vesicles Secreted in Response to Cytokine Exposure Increase Mitochondrial Oxygen Consumption in Recipient Cells. Front Cell Neurosci (2019) 13:1–12. 10.3389/fncel.2019.00051 30837842 PMC6383587

[94] Bland CL Byrne-Hoffman CN Fernandez A Rellick SL Deng W Klinke DJ. Exosomes derived from B16F0 melanoma cells alter the transcriptome of cytotoxic T cells that impacts mitochondrial respiration. FEBS J (2018) 285:1033–50. 10.1111/febs.14396 PMC6457271 29399967

[95] Yao J Zheng J Cai J Zeng K Zhou C Zhang J. Extracellular vesicles derived from human umbilical cord mesenchymal stem cells alleviate rat hepatic ischemia-reperfusion injury by suppressing oxidative stress and neutrophil inflammatory response. FASEB J (2019) 33:1695–710. 10.1096/fj.201800131RR 30226809

[96] Li H Xia Z Chen Y Qi D Zheng H. Mechanism and Therapies of Oxidative Stress-Mediated Cell Death in Ischemia Reperfusion Injury. Oxid Med Cell Longev (2018) 2018:1–2. 10.1155/2018/2910643 PMC6035842 30034574

[97] Wang S-Y Hong Q Zhang C-Y Yang Y-J Cai G-Y Chen X-M. miRNAs in stem cell-derived extracellular vesicles for acute kidney injury treatment: comprehensive review of preclinical studies. Stem Cell Res Ther (2019) 10:281. 10.1186/s13287-019-1371-1 31481100 PMC6724288

[98] Gu D Zou X Ju G Zhang G Bao E Zhu Y. Mesenchymal Stromal Cells Derived Extracellular Vesicles Ameliorate Acute Renal Ischemia Reperfusion Injury by Inhibition of Mitochondrial Fission through miR-30. Stem Cells Int (2016) 2016:2093940. 10.1155/2016/2093940 27799943 PMC5069372

[99] Cao H Cheng Y Gao H Zhuang J Zhang W Bian Q. In Vivo Tracking of Mesenchymal Stem Cell-Derived Extracellular Vesicles Improving Mitochondrial Function in Renal Ischemia-Reperfusion Injury. ACS Nano (2020) 14:4014–26. 10.1021/acsnano.9b08207 32212674

[100] Gallo S Gili M Lombardo G Rossetti A Rosso A Dentelli P. Stem Cell-Derived, microRNA-Carrying Extracellular Vesicles: A Novel Approach to Interfering with Mesangial Cell Collagen Production in a Hyperglycaemic Setting. PloS One (2016) 11:1–18. 10.1371/journal.pone.0162417 PMC5017750 27611075

[101] Gomez IG MacKenna DA Johnson BG Kaimal V Roach AM Ren S. Anti-microRNA-21 oligonucleotides prevent Alport nephropathy progression by stimulating metabolic pathways. J Clin Invest (2015) 125:141–56. 10.1172/JCI75852 PMC4382246 25415439

[102] Wang K Jiang Z Webster KA Chen J Hu H Zhou Y. Enhanced Cardioprotection by Human Endometrium Mesenchymal Stem Cells Driven by Exosomal MicroRNA-21. Stem Cells Trans Med (2017) 6:209–22. 10.5966/sctm.2015-0386 PMC5442741 28170197

[103] Zhu J Lu K Zhang N Zhao Y Ma Q Shen J. Myocardial reparative functions of exosomes from mesenchymal stem cells are enhanced by hypoxia treatment of the cells via transferring microRNA-210 in an nSMase2-dependent way. Artif Cells Nanomed Biotechnol (2018) 46:1659–70. 10.1080/21691401.2017.1388249 PMC5955787 29141446

[104] Hashimoto H Olson EN Bassel-Duby R. Therapeutic approaches for cardiac regeneration and repair. Nat Rev Cardiol (2018) 15:585–600. 10.1038/s41569-018-0036-6 29872165 PMC6241533

[105] Lv G Shao S Dong H Bian X Yang X Dong S. MicroRNA-214 protects cardiac myocytes against H2O2-induced injury. J Cell Biochem (2014) 115:93–101. 10.1002/jcb.24636 23904244

[106] Wang Y Zhao R Liu D Deng W Xu G Liu W. Exosomes Derived from miR-214-Enriched Bone Marrow-Derived Mesenchymal Stem Cells Regulate Oxidative Damage in Cardiac Stem Cells by Targeting CaMKII. Oxid Med Cell Longev (2018) 2018:4971261. 10.1155/2018/4971261 30159114 PMC6109555

[107] Shi B Wang Y Zhao R Long X Deng W Wang Z. Bone marrow mesenchymal stem cell-derived exosomal miR-21 protects C-kit+ cardiac stem cells from oxidative injury through the PTEN/PI3K/Akt axis. PloS One (2018) 13:e0191616. 10.1371/journal.pone.0191616 29444190 PMC5812567

[108] Warburg O Wind F Negelein E. THE METABOLISM OF TUMORS IN THE BODY. J Gen Physiol (1927) 8:519–30. 10.1085/jgp.8.6.519 PMC2140820 19872213

[109] Potter M Newport E Morten KJ. The Warburg effect: 80 years on. Biochem Soc Trans (2016) 44:1499–505. 10.1042/BST20160094 PMC5095922 27911732

[110] Gu Z-W He Y-F Wang W-J Tian Q Di W. MiR-1180 from bone marrow-derived mesenchymal stem cells induces glycolysis and chemoresistance in ovarian cancer cells by upregulating the Wnt signaling pathway. J Zhejiang Univ Sci B (2019) 20:219–37. 10.1631/jzus.B1800190 PMC6421125 30829010

[111] Eom YW Shim KY Baik SK. Mesenchymal stem cell therapy for liver fibrosis. Korean J Intern Med (2015) 30:580–9. 10.3904/kjim.2015.30.5.580 PMC4578027 26354051

[112] Alfaifi M Eom YW Newsome PN Baik SK. Mesenchymal stromal cell therapy for liver diseases. J Hepatol (2018) 68:1272–85. 10.1016/j.jhep.2018.01.030 29425678

[113] Esau C Davis S Murray SF Yu XX Pandey SK Pear M. miR-122 regulation of lipid metabolism revealed by in vivo antisense targeting. Cell Metab (2006) 3:87–98. 10.1016/j.cmet.2006.01.005 16459310

[114] Lou G Yang Y Liu F Ye B Chen Z Zheng M. MiR-122 modification enhances the therapeutic efficacy of adipose tissue-derived mesenchymal stem cells against liver fibrosis. J Cell Mol Med (2017) 21:2963–73. 10.1111/jcmm.13208 PMC5661245 28544786

[115] Elhanati S Ben-Hamo R Kanfi Y Varvak A Glazz R Lerrer B. Reciprocal Regulation between SIRT6 and miR-122 Controls Liver Metabolism and Predicts Hepatocarcinoma Prognosis. Cell Rep (2016) 14:234–42. 10.1016/j.celrep.2015.12.023 26748705

